# Prevalence of Underlying Diseases in Hospitalized Patients with COVID-19: a Systematic Review and Meta-Analysis 

**Published:** 2020-03-24

**Authors:** Amir Emami, Fatemeh Javanmardi, Neda Pirbonyeh, Ali Akbari

**Affiliations:** 1 Microbiology Department, Burn and Wound Healing Research Center, Shiraz University of Medical Sciences, Shiraz, Iran.; 2 Department of Anesthesiology, School of Medicine, Shiraz University of Medical Sciences, Shiraz, Iran.

**Keywords:** Comorbidity, COVID-19, severe acute respiratory syndrome coronavirus 2, Meta-analysis

## Abstract

**Introduction:**

In the beginning of 2020, an unexpected outbreak due to a new corona virus made the headlines all over the world. Exponential growth in the number of those affected makes this virus such a threat. The current meta-analysis aimed to estimate the prevalence of underlying disorders in hospitalized COVID-19 patients.

**Methods:**

A comprehensive systematic search was performed on PubMed, Scopus, Web of science, and Google scholar, to find articles published until 15 February 2020. All relevant articles that reported clinical characteristics and epidemiological information of hospitalized COVID-19 patients were included in the analysis.

**Results:**

The data of 76993 patients presented in 10 articles were included in this study. According to the meta-analysis, the pooled prevalence of hypertension, cardiovascular disease, smoking history and diabetes in people infected with SARS-CoV-2 were estimated as 16.37% (95%CI: 10.15%-23.65%), 12.11% (95%CI 4.40%-22.75%), 7.63% (95%CI 3.83%-12.43%) and 7.87% (95%CI 6.57%-9.28%), respectively.

**Conclusion:**

According to the findings of the present study, hypertension, cardiovascular diseases, diabetes mellitus, smoking, chronic obstructive pulmonary disease (COPD), malignancy, and chronic kidney disease were among the most prevalent underlying diseases among hospitalized COVID-19 patients, respectively.

## Introduction

In late 2019, a novel corona virus (first: 2019-nCov, then: SARS-CoV-2) was identified as the cause of a cluster of pneumonia cases, which infected a lot of people in Wuhan, a city in the Hubei province of China (1). SARS-CoV-2 rapidly spread and led to an outbreak in China and then became a global health emergency. Although control measures and isolations have been applied for prevention, the infection has increased and caused a pandemic (2). Although this virus belongs to a relatively well-known viral family, Coronaviridae, and is similar to viruses that caused severe acute respiratory syndrome (SARS), which had an outbreak in 2002, and Middle East respiratory syndrome (MERS), which had an outbreak in 2012, in some characteristics, there are a lot of uncertainties and unknown specifications about this virus such as its origin and source of infection, its emergence, and its mechanism of action and transmission (3, 4). 

Since the number of COVID-2019 cases is rising around the world and it has been associated with a large number of mortality and morbidity, it has led to a new global phobia called Coro phobia (5). Based on recent reports, the novel Corona virus can be identified through various symptoms (Fever, Cough, Dyspnea, Myalgia, and Fatigue) (6-8). 

Similar to other viral respiratory infections, SARS-CoV-2 or COVID-19 can be transmitted through the respiratory tract. It mainly causes respiratory tract infections and develops severe pneumonia in infected patients who may require intensive care. Severe disease may result in death due to progressive respiratory failure (9, 10).

Everyone is susceptible to this virus, but the elderly and those with underlying diseases are more at risk of adverse outcomes. Current knowledge has shown that death rate is high in people with chronic underlying diseases (11). Therefore, special attention should be paid to the elderly and immunocompromised patients. Infections might progress rapidly in these groups and timely clinical decisions are needed (12). Currently, information on the prevalence of predominant chronic diseases is rare. Moreover, knowing the underlying diseases in COVID-19 infected patients is important for healthcare workers. In the current study, a systematic review and meta-analysis was conducted on the prevalence of underlying diseases in confirmed hospitalized COVID-19 cases. 

## Methods

**Search Strategy**

In order to find relevant studies, international databases including PubMed, Scopus, Web of Science, Google scholar, and Embase were searched for articles published until 16 February 2020. The following search terms were used (designed using English MeSH keywords and Emtree terms): [SARS-CoV-2 AND characteristics] OR , [2019-nCoV AND Characteristics]” OR “COVID-19 AND Comorbidities] OR [new coronavirus AND Characteristics AND Comorbidities] OR [Wuhan Coronavirus AND Characteristics AND Comorbidities] OR [Coronavirus AND characteristics AND Comorbidities]. Additionally, extra searches were performed in the reference lists of included studies to avoid missing papers. Moreover Centers for Disease Control and Prevention (CDC) and World Health Organization (WHO) portals as the national public health institute were evaluated. Due to the huge number of articles in Chinese language, the abstracts were evaluated in these studies.

**Inclusion and Exclusion Criteria**

Any relevant articles that reported clinical characteristics and epidemiological information on infected patients were included in the analysis. All articles with any design (randomized controlled trials, non-randomized controlled trials, case-control studies, cross-sectional studies) were included. Articles were excluded if appropriate information was not reported.

**Data extraction and paper quality evaluation**

Two authors (A.E. and F.J.) screened and evaluated the literature independently. All the included papers were assessed using the Newcastle-Ottawa Scale and the results are provided in table 1 (13). The following features were extracted for pooled estimation: name of the first authors and age, sex, and coexisting condition of the patients.

**Statistical analysis**

Overall prevalence with 95% confidence interval was estimated via inverse variance method. Heterogeneity was evaluated using chi-square and *I*^2^. The random effect model was used in case of considerable heterogeneity, which was defined as I²>75%. Sensitivity analysis was done according to outlier data. Egger’s regression test was used to evaluate publication biases. All statistical analyses were performed using STATA 13, metaprop command. 

## Results

***Characteristics of included studies***

**Figure 1:**
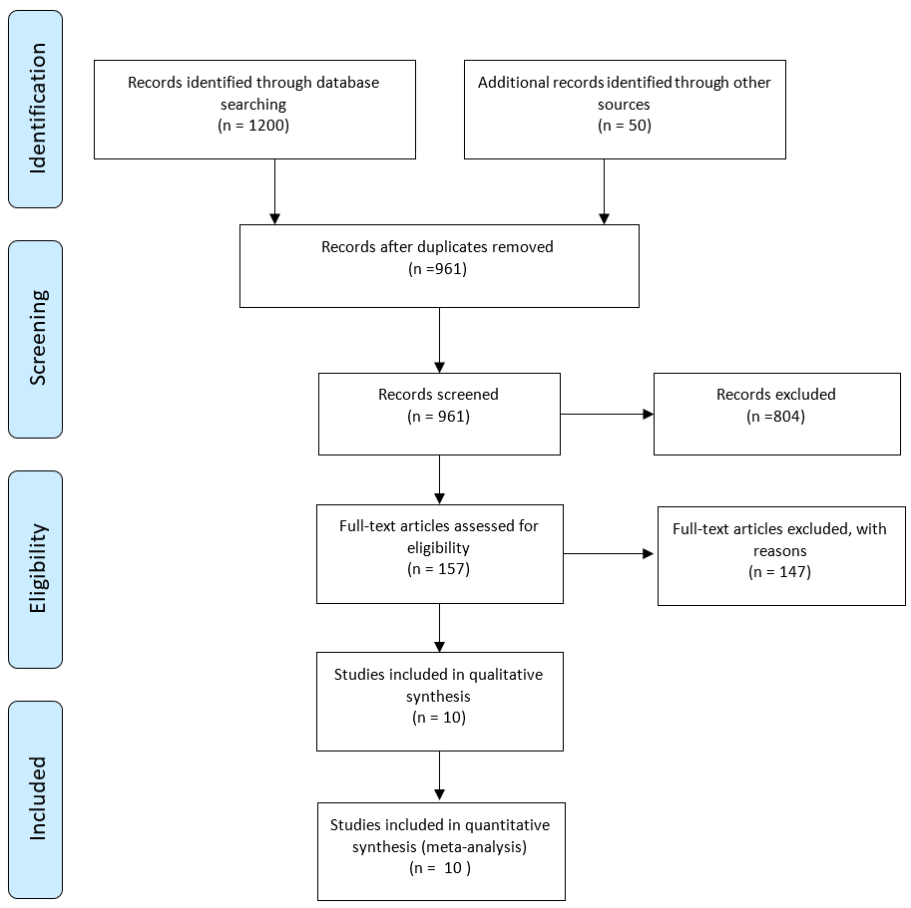
PRISMA flow chart of the systematic literature review and article identification.

In the initial search, 1250 articles were found in different databases. All papers were screened by reading their abstracts and 289 of them were eliminated due to being duplicates found in different databases. After evaluating the full texts, 804 studies were excluded due to presenting data that were irrelevant to our aim. 10 articles met the inclusion criteria but some of the required information was not reported in all of the articles. Figure1 shows the search details, and the characteristics of included studies are provided in table 2. Finally, the available data of 3,403 hospitalized patients with COVID-19 infection were used for the analysis.

***Prevalence of underlying diseases in hospitalized COVID-19 cases***

**- Hypertension**

**Figure 2:**
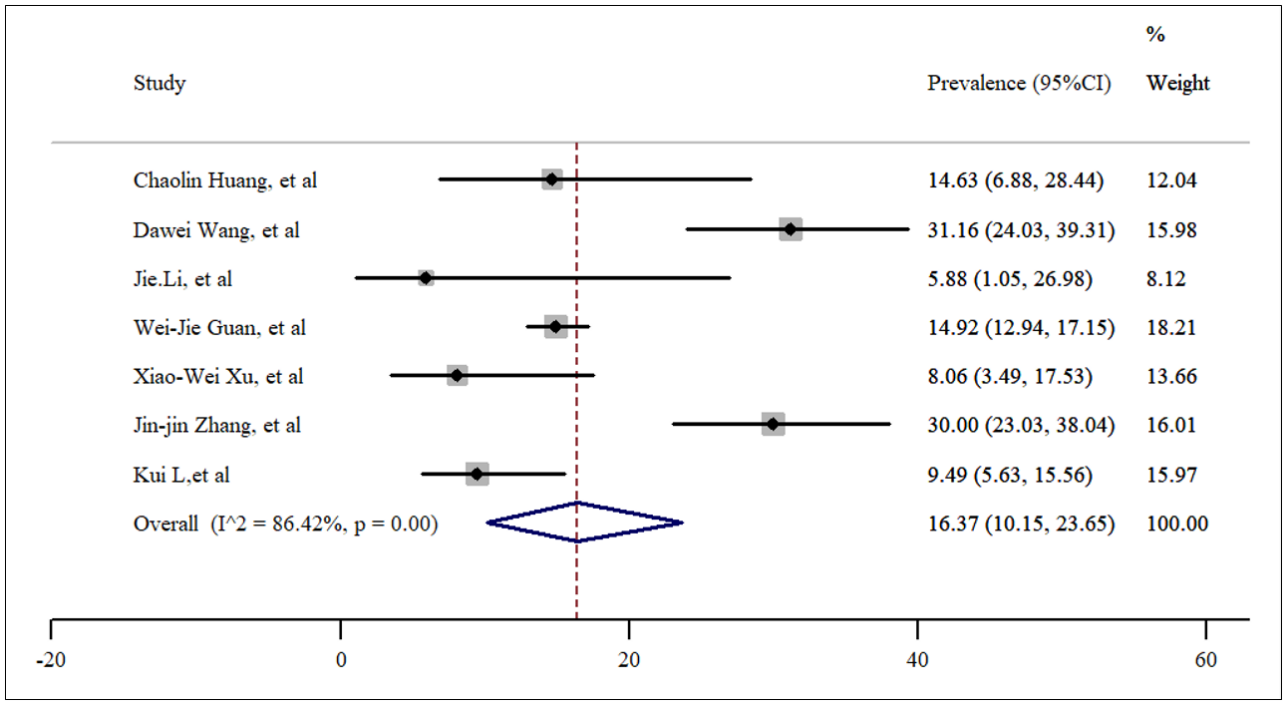
Prevalence of hypertension among patients hospitalized with COVID-19.

**Figure 3:**
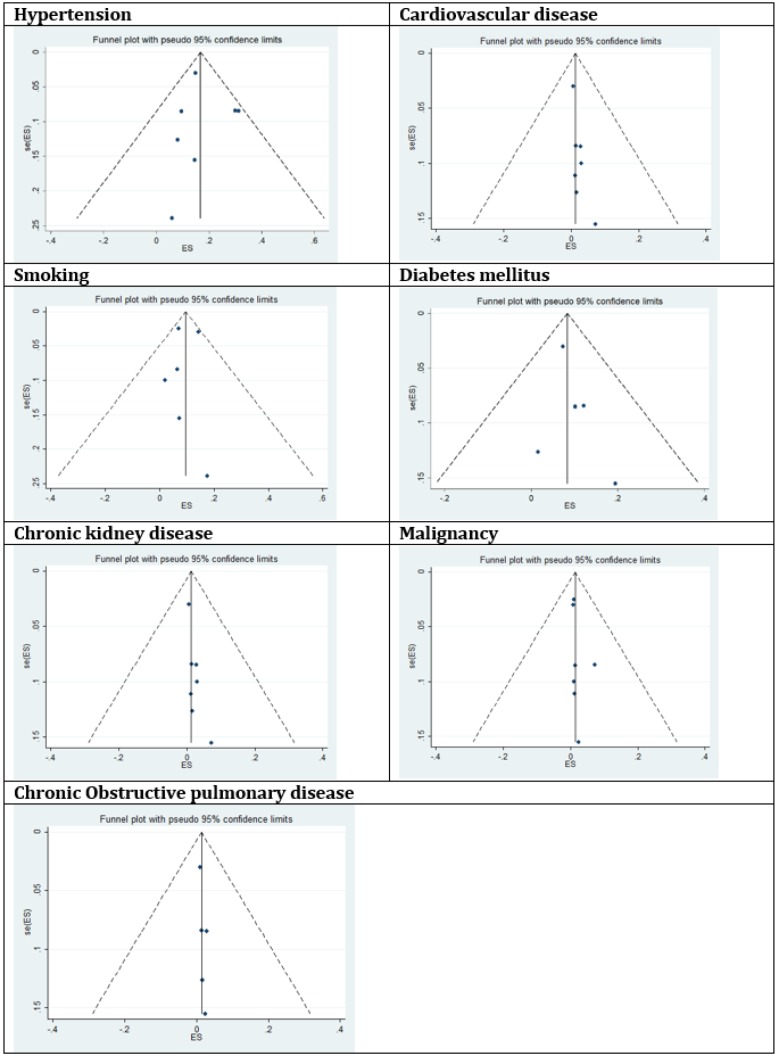
Funnel plot for meta-analysis of the prevalence of underlying diseases in COVID-19 infected cases.

Through the current meta-analysis, it was found that hypertension is the most prevalent underlying disease in hospitalized COVID-19 cases. 16% (95%: CI: 10.15%-23.65%) of SARS-CoV-2 infected cases were hypertensive (figure 2). This information was reported in 7 studies. According to the *I*^2^ index, which was calculated to be 86.42%, and the Chi-square results, there was high and significant heterogeneity between the studies (P<0.001). No publication bias was found in studies (t= -1.67, P=0.15). In addition, the corresponding funnel plot is provided in figure 3.

**- Cardiovascular disease**

In order to estimate the pooled prevalence of cardiovascular disease in COVID-19 patients, 8 studies were evaluated. The incidence was 12.11% (95%CI: 4.40% – 22.75%), with high and significant heterogeneity (*I*^2^=95.89%), also no publication bias was present according to Eggers’s test (t= 1.99, p=0.09). The funnel plot has been shown in figure 3. Sensitivity analysis did not show significant changes. 

**- Smoking**

**Figure 4:**
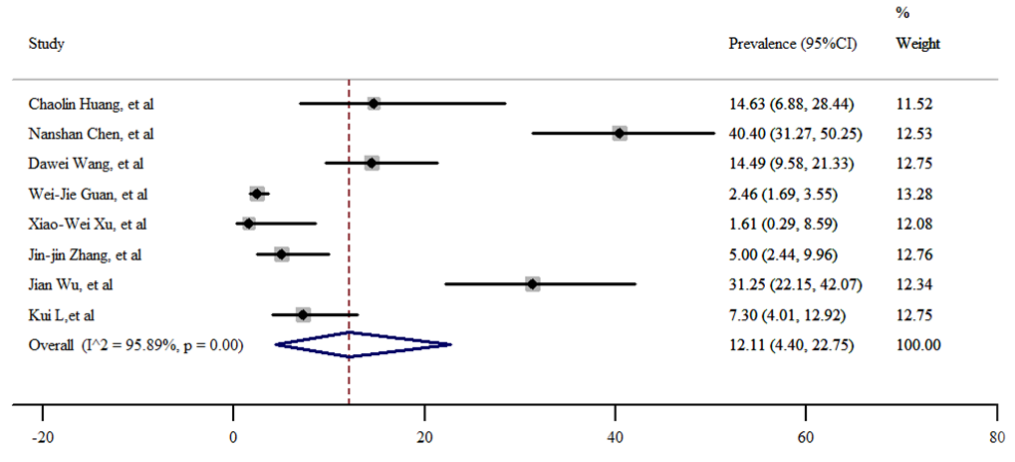
Prevalence of cardiovascular disease among patients hospitalized with COVID-19.

In the forest plot drawn (Figure 4), the pooled prevalence of SARS-CoV-2 infection in hospitalized patients with history of smoking was estimated as 7.63 percent. High and significant heterogeneity was found between the 6 included studies (*I*^2^= 90.19%, p < 0.001). Moreover, no publication bias was found (t= -0.24, p=0.82). It is worth noting that the number of smokers in Wenhua Liang’s study was calculated using the information presented in the study (14). 

**- Diabetes mellitus**

**Figure 5:**
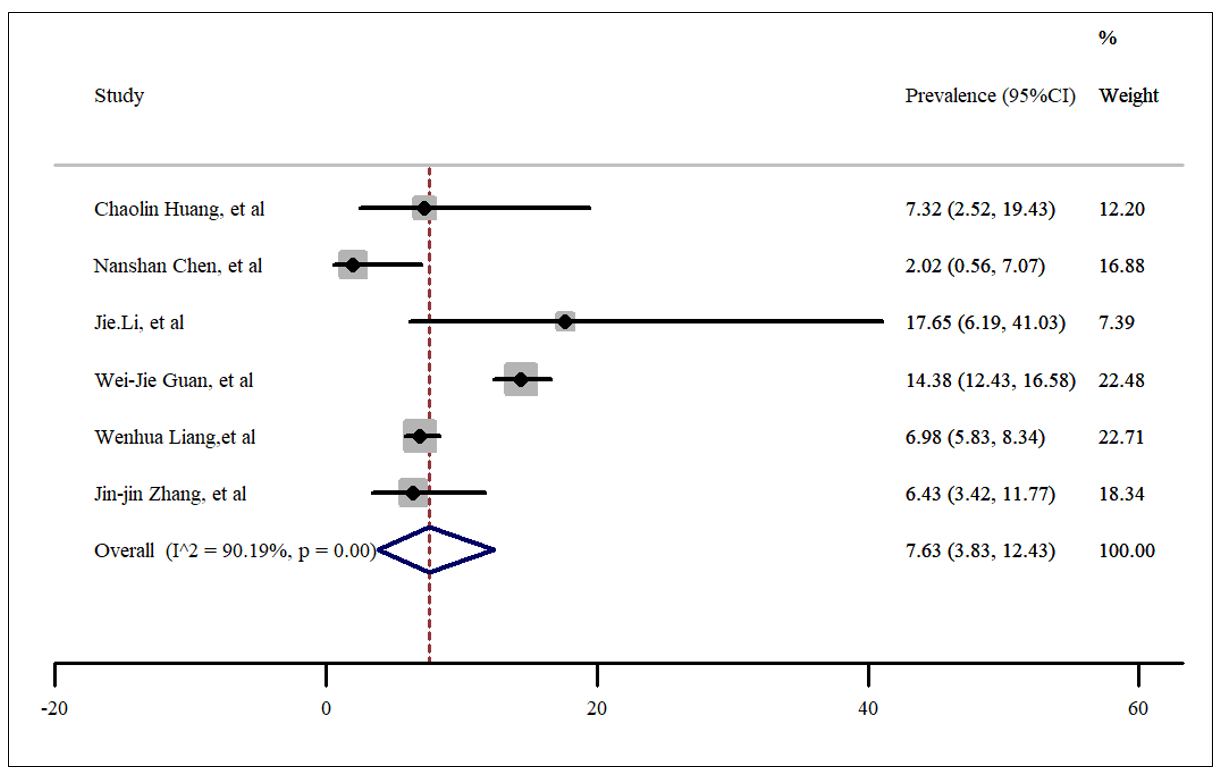
Prevalence of smokers among patients hospitalized with COVID-19.

Using the data of 6 included articles, the prevalence of diabetes among people who were infected with SARS-CoV-2 was estimated to be 7.87% (95%CI: 6.57% – 9.28%), which is presented in figure 5. No publication bias was present based on Egger’s test and the funnel plot presented in figure 3 (t=-1.64, p=0.17).

**- Chronic kidney disease**

**Figure 6:**
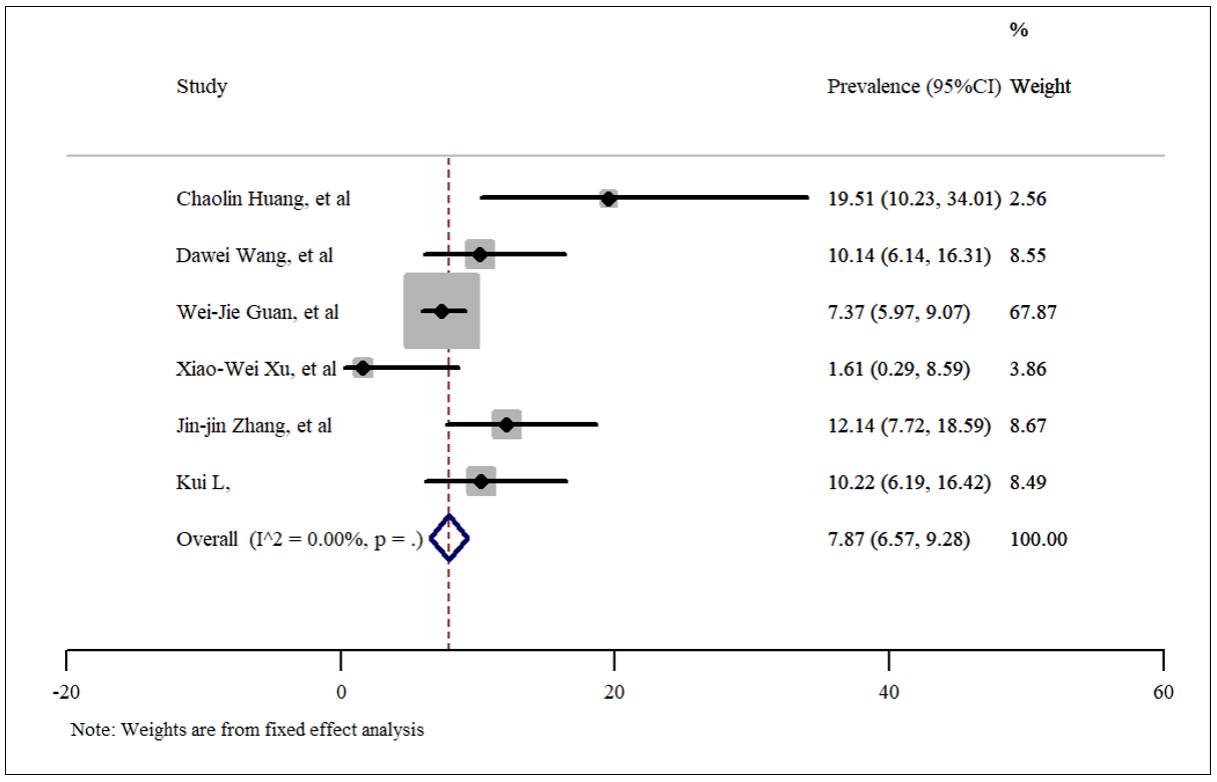
Prevalence of diabetes among patients hospitalized with COVID-19.

As shown in figure 6, the pooled prevalence of acute kidney diseases in SARS-CoV-2 hospitalized patients was estimated as 0.83% (95% CI: 0.37%- 1.43%). A fixed model was used for meta-analysis of data presented in 7 included studies. No publication bias was present according to Egger’s test and the related funnel plot presented in figure 3 (t=1.83, p=0.12)

**- Malignancy**

**Figure 7:**
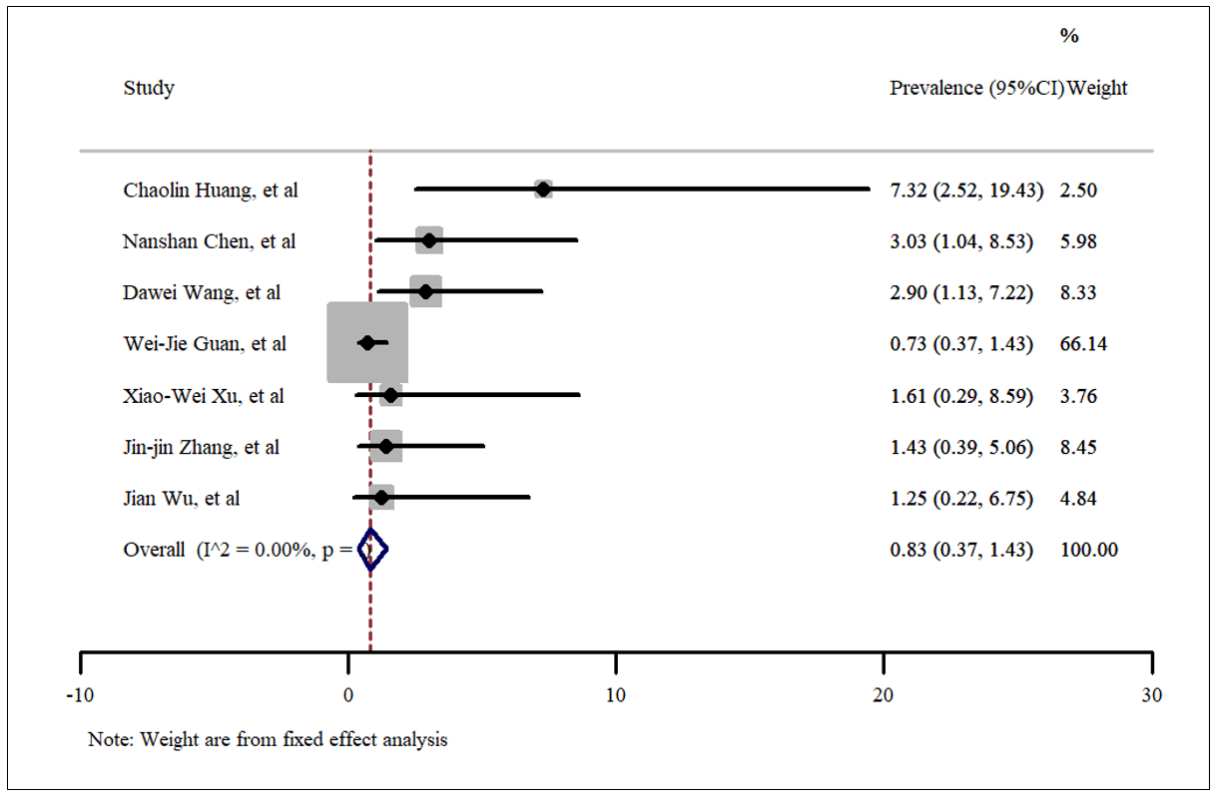
Prevalence of kidney disease among patients hospitalized with COVID-19.

The pooled prevalence of malignancy among hospitalized COVID-19 patients was estimated to be 0.92% (95% CI: 0.56%-1.34%) (results presented in 7 articles, Figure 7). In this section, a fixed effect analysis was used. The value of t in Egger’s test was 1.14 and the p-value was 0.305, which means that no publication bias was present. The related funnel plot is depicted in figure 3. 

**- Chronic obstructive pulmonary diseases (COPD)**

**Figure 8:**
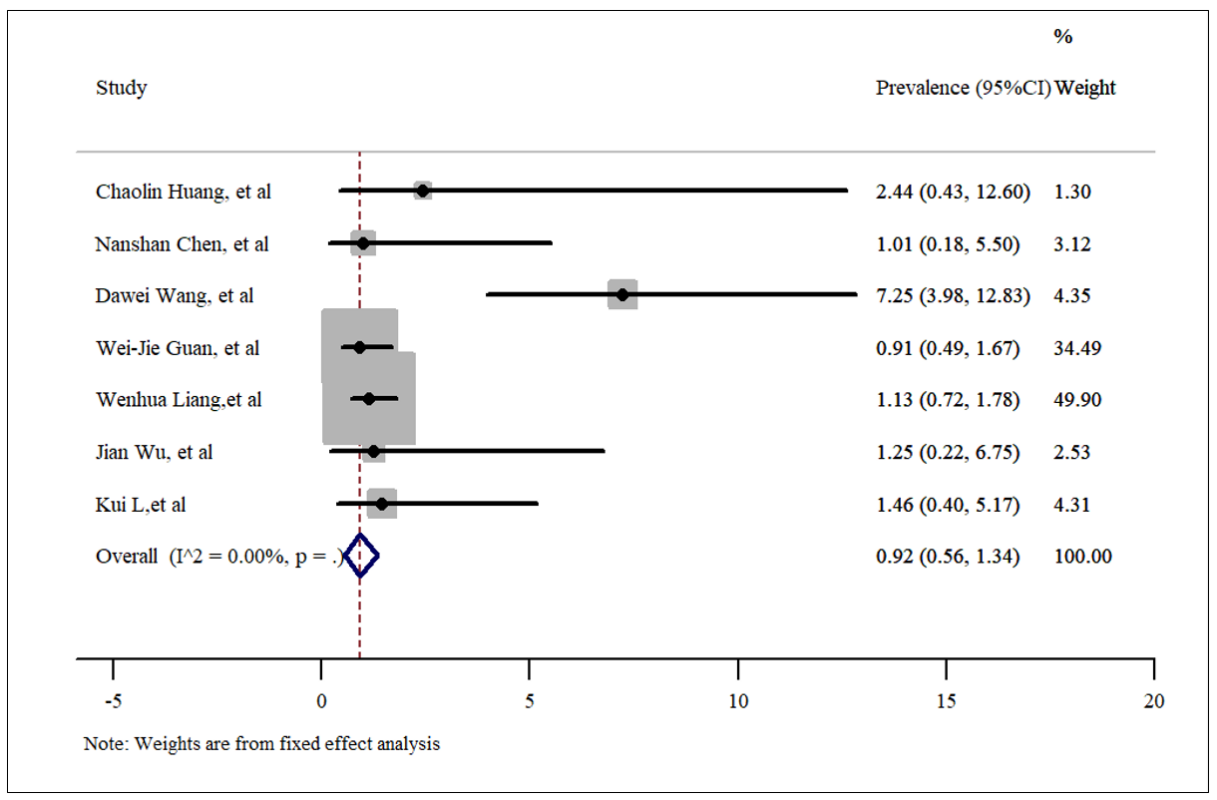
Prevalence of malignancies among patients hospitalized with COVID-19.

The last comorbidity that was studied using the included articles was chronic obstructive pulmonary diseases (COPD). According to our statistical analysis, the incidence rate of COPD in hospitalized COVID-19 patients was 0.95% (95% CI: 0.43%-1.61%). Although there were many articles about this new Coronavirus, this coexisting disorder was reported in only 5 published studies. In order to evaluate the pooled prevalence, a fixed model was used and the results are shown in Figure 8. The value of t in Egger’s test was found to be 1.81 and the p-value was 0.16, which means that no publication bias was present. The related funnel plot is shown in figure 3. 

## Discussion

China and the rest of the world have faced an outbreak of a novel Corona virus. The widespread distribution of this virus has led to a major concern, globally. Human coronaviruses are among the pathogens causing viral respiratory infections, and the recently detected strain called SARS-CoV-2 has caused a big challenge for countries all over the world (15, 16). This is the third contagious Coronavirus leading to an epidemic in the 21^st^ century after MERS and SARS (17). The key problems surrounding this novel virus are as follows: diagnosis, mode of transmission, long incubation period (3 to 14 days), predicting the number of infected cases in the community, and insufficient protection resources due to its pandemic specification (15, 18). The accurate transmission rate of SARS-CoV-2 is unknown, since various factors impact its transmission. Moreover, infection of family clusters and healthcare workers indicate the human to human transmission of the disease and its contagiousness, which makes the condition more complicated (19, 20). 

Since SARS-CoV-2 is a newly identified pathogen, there is no pre-existing immunity to it in the human community, also there is no definitive cure to interrupt or reduce its astonishing spread. These ambiguities make the condition more serious for vulnerable members of the community, which include individuals with immune problems, co-existing comorbidity and elderly people. Despite the novelty of the topic, there are a lot of proposed studies about history, transmission route, urgency of responding, pathogenic potential characteristics and prevention strategies but there are still some underlying diseases that have remained unknown (21).

**Table 1:** NEWCASTLE-OTTAWA quality assessment scale for cross sectional studies

**First author, year**	**Selection**	**Comparability**	**Exposure/Outcome**	**Total Score**
Chaolin Huang, 2020	*****	*	***	*********
Nanshan Chen,2020	*****	**	***	**********
Dawei Wang, 2020	*****	**	***	**********
Jie.Li, 2020	*****	**	***	**********
Wei-Jie Guan, 2020	*****	**	***	**********
Xiao-Wei Xu, 2020	*****	*	**	********
Wenhua Liang,2020	*****	*	***	*********
Jin-jin Zhang, 2020	*****	**	***	**********
Jian Wu, 2020	*****	**	***	**********
Kui L2020	****	**	***	*********

**Table 2:** characteristics of included studies

**First Author **	**n**	**Sex (M/F)**	**Age**	**CKD **	**HTN**	**DM**	**Mal**	**Smoking**	**COPD**	**CVD**
Chaolin Huang, et al. (34)	41	30/11	Range: 41-58	3	6	8	1	3	1	6
Nanshan Chen, et al. (2)	99	67/32	Mean: 55.5 ±13.1	3			1	2		40
Dawei Wang, et al. (35)	138	78/63	Median: 56 (42-68)	4	43	14	10		4	20
Jie.Li, et al. (36)	17	9/8	Range: 22-65	8	1			3		
Wei-Jie Guan, et al. (37)	1099	640/459	Range: 35-58	1	164	81	10	158	12	27
Xiao-Wei Xu, et al. (38)	62	35/27	Median: 41 (32-52)		5	1			1	1
Wenhua Liang,et al. (14)	1590	-	-				18	111		
Jin-jin Zhang, et al. (16)	140	71/69	Median: 57 (25-87)	2	42	17		9	2	7
Jian Wu, et al. (39)	80	39/41	Mean: 46.1 ± 15.4	1			1			25
Kui L, et al. (40)	137	61/76	Median: 57(20-83)		13	14	2			10

CKD: chronic kidney disease; HTN: hypertension; DM: diabetes mellitus; Mal: malignancy; COPD: chronic obstructive pulmonary diseases; CVD: cardiovascular disorders.

**Figure 9:**
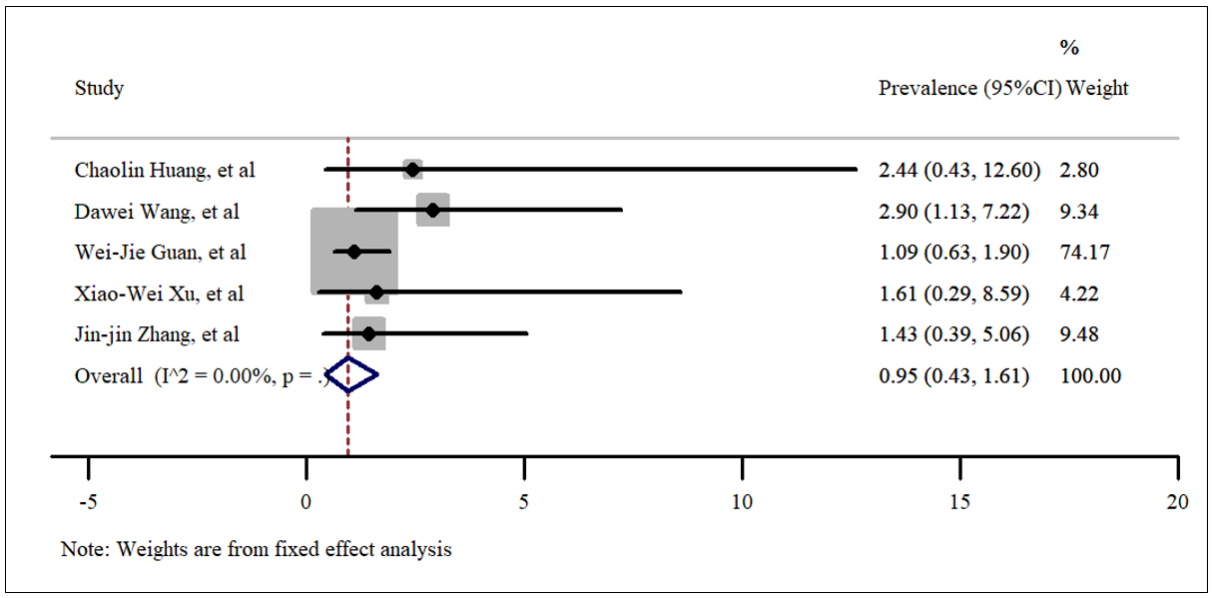
Prevalence of chronic obstructive pulmonary disease (COPD) among patients hospitalized with COVID-19.

According to the current analysis, hypertension, cardiovascular diseases, diabetes, kidney disease, smoking, and COPDs were among the most prevalent underlying diseases among hospitalized patients with COVID-19.

In terms of pre-existing medical conditions, cardiovascular diseases had the highest prevalence among diseases that put patients at higher risk of SARS-CoV-2 threats. Decreasing the pro-inflammatory cytokines, which leads to a weaker immune function may account for this condition (2, 22). It is worth noting that similar results were found regarding MERS (23). We also found that smokers are more susceptible to Coronavirus infections, especially to the most recent species. Various reasons may justify this happening. It has been mentioned that smokers have unregulated ACE2 in remodeled cell types, which is consistent with results of SARS studies. However, factors such as amount of smoking, the duration of smoking, and the duration of smoking cessation also play a role. In some previous studies on MERS-CoV-2 it has been shown that dipeptidyl peptidase IV (DPP4), which is the specific receptor for this virus, had a higher rate of expression in smokers and COPD patients (24). 

Although the results of the current analysis indicate that smoking can be an underlying factor that makes people susceptible to COVID-19 complications, in some studies, especially COVID-19 related studies, no strong evidence has been found regarding the correlation of COPD and smoking with being infected with this new virus. But the important point that must be taken into consideration is that the outcome of SARS-CoV-2 infection is more severe in COPD cases and smokers (25). 

As mentioned in the results section, patients with malignancies are more in danger than those without any tumor. Anticancer treatments such as chemotherapy and surgery put this group into an immunosuppressive state and subsequently at higher risk of MERS-CoV-2 infection (26). Among those with malignancies, lung cancer patients seems to be more susceptible, and they must follow guidance on restricting any contact with possible infected zones or individuals for their safety (14).

Possible risk factors for progressive and severe illness may include the above-mentioned factors but are not limited to them; pregnancy and old age are other risky conditions, which should be monitored meticulously. However, there is no clear evidence about the risk of transmission of COVID-19 to the newborn during vaginal delivery or transmission via breastfeeding but care and protection of newborns against possible exposure to infection or contaminated conditions such as maternal breast contamination must be observed. Since MERS-CoV-2 is an emerging virus, no specific treatment is currently available (27, 28), and pathophysiology of this condition is still unknown. Therefore, general prevention measures such as the following should be followed: Washing hands frequently and avoiding touching the eyes, nose, and mouth with contaminated hands, avoiding close contact ,especially with those who have fever, coughing or sneezing, avoiding contact with live animals and consuming raw animal products (29). 

There are some responsibilities for health policymakers in this critical condition: Screening of travelers, triage all patients on admission and immediately isolating all suspected and confirmed cases, providing protective gear, preparing local guidance and instructions for people, especially for high risk groups (30, 31). 

To the best of our knowledge, this is the first meta-analysis that estimates the prevalence of underlying diseases in patients infected with SARS-CoV-2. Given that most studies on CoVID-19 are in an early stage, and there are some limitations such as small number of studies, and reports being restricted to China and a few other countries, due to the pandemic nature of the disease, specific patterns should be introduced for different groups, including people with underlying diseases, to minimize the harm.

Based on the experiences gained on this disease during this short time, a strong recommendation for all people, clinicians, and policymakers is to guide people to protect themselves to avoid being exposed to SARS-CoV-2, whenever possible (32). Another very important advice to patients with underlying diseases during the epidemics like the one caused by the novel virus is to follow guidance on travel restrictions. These groups must be aware of their high-risk situation and comply with all health guidelines such as hand hygiene, face care, and restricting social interactions. In addition, to reduce the morbidity and complications of COVID-19 in different populations, especially patients with the mentioned underlying diseases, we recommend clinicians and policymakers to launch diagnostic procedures for such individuals first so that proper treatments can be designed and followed to ensure they are protected within epidemic regions (33). 

In summary, the results of the current study have shown that in patients with SARS-CoV-2 infection, hypertension, cardiovascular disease, smoking, and diabetes are the most prevalent co-existing disorders. Given that COVID-19 has a relatively long incubation period and during this time the infected person can transmit the virus without showing symptoms, it is strongly recommended that patients with chronic or underlying diseases avoid any close contact with other people in the community, especially in epidemic areas. During the current SARS-CoV-2 pandemic, the statistics reported by different countries regarding associated mortality of those with risk factors, incubation time, and estimated overall mortality have not been consistent and general conclusions should be drawn with caution. It should be noted that the outbreak worsens with decrease in adherence to diagnostic guidelines and prevention strategies, such as avoiding traveling and gathering in public places. 

## Conclusion

According to the findings of the present study, hypertension, cardiovascular diseases, diabetes mellitus, smoking, COPD, malignancy, and chronic kidney disease were among the most prevalent underlying diseases among hospitalized patients with COVID-19, respectively. 
